# Autoregulatory function of interleukin-10-producing pre-naïve B cells is defective in systemic lupus erythematosus

**DOI:** 10.1186/s13075-015-0687-1

**Published:** 2015-07-25

**Authors:** Ji Hyun Sim, Hang-Rae Kim, Soog-Hee Chang, In Je Kim, Peter E. Lipsky, Jisoo Lee

**Affiliations:** Department of Anatomy, Seoul National University College of Medicine, Daehak-ro 103, Seoul, 110-799 Republic of Korea; Department of Biomedical Sciences, Seoul National University College of Medicine, Daehak-ro 103, Seoul, 110-799 Republic of Korea; Division of Rheumatology, Ewha Womans University School of Medicine, Anyangcheon-gil 1071, Seoul, 158-710 Korea; 1545 London Road, Charlottesville, VA 20901 USA

## Abstract

**Introduction:**

Pre-naïve B cells represent an intermediate stage in human B-cell development with some functions of mature cells, but their involvement in immune responses is unknown. The aim of this study was to determine the functional role of normal pre-naïve B cells during immune responses and possible abnormalities in systemic lupus erythematosus (SLE) that might contribute to disease pathogenesis.

**Methods:**

Pre-naïve, naïve, and memory B cells from healthy individuals and SLE patients were stimulated through CD40 and were analyzed for interleukin-10 (IL-10) production and co-stimulatory molecule expression and their regulation of T-cell activation. Autoreactivity of antibodies produced by pre-naïve B cells was tested by measuring immunoglobulin M (IgM) autoantibodies in culture supernatants after differentiation.

**Results:**

CD40-stimulated pre-naïve B cells produce larger amounts of IL-10 but did not suppress CD4^+^ T-cell cytokine production. Activated pre-naïve B cells demonstrated IL-10-mediated ineffective promotion of CD4^+^ T-cell proliferation and induction of CD4^+^FoxP3^+^ T cells and IL-10 independent impairment of co-stimulatory molecule expression and tumor necrosis factor-alpha (TNF-α) and IL-6 production. IgM antibodies produced by differentiated pre-naïve B cells were reactive to single-stranded deoxyribonucleic acid. SLE pre-naïve B cells were defective in producing IL-10, and co-stimulatory molecule expression was enhanced, resulting in promotion of robust CD4^+^ T-cell proliferation.

**Conclusions:**

There is an inherent and IL-10-mediated mechanism that limits the capacity of normal pre-naïve B cells from participating in cellular immune response, but these cells can differentiate into autoantibody-secreting plasma cells. In SLE, defects in IL-10 secretion permit pre-naïve B cells to promote CD4^+^ T-cell activation and may thereby enhance the development of autoimmunity.

**Electronic supplementary material:**

The online version of this article (doi:10.1186/s13075-015-0687-1) contains supplementary material, which is available to authorized users.

## Introduction

B-cell maturation in adults occurs in steps. First, in the bone marrow, stem cells undergo a series of precursor stages during which they rearrange their immunoglobulin (Ig) genes to generate a wide range of unique antigen-binding specificities to develop into immature/transitional B cells. Then, in the periphery, they mature from transitional to fully mature naïve B cells. Each developmental step is tightly controlled by the expression and function of the B-cell receptor (BCR) [1]. In mice, transitional B cells can be subdivided into two developmental subsets, T1 and T2, based on expression of CD21 and IgD. CD24^hi^CD21^lo^IgD^lo^ T1 and CD24^hi^CD21^hi^IgD^hi^ T2 cells appear to have different population dynamics, and require different maturation signals [2]. This multistep development process during the maturation from transitional B cells into naïve B cells has also been identified recently in humans. Based on CD38 expression levels, human peripheral blood immature B cells could be subdivided into CD27^−^CD38^hi^IgD^+^ transitional B cells and CD27^−^CD38^int^IgD^+^ pre-naïve B cells [3, 4]. The comprehensive phenotyping and initial functional analysis clearly demonstrated that pre-naïve B cells were a maturation intermediate between transitional and naïve B cells with unique properties and functions. Notably, human peripheral maturational B-cell subsets, including pre-naïve B cells, express CD5, whereas in mice, CD5 is expressed on specialized B-cell subset B-1 B cells [3, 5].

The essential role of mature B cells is the production of antigen (Ag)-specific antibodies (Abs) during humoral immunity by differentiating into plasma cells [6]. B cells also mediate many other functions essential for immune homeostasis. B cells are required for initiation of T-cell immune responses by presenting Ags, providing co-stimulation, and producing cytokines to activate and expand effectors and memory T-cell populations [7]. In addition, B cells can negatively regulate immune responses by directly inhibiting CD4^+^ T cells and by inducing regulatory T cells (Tregs) through production of the cytokine interleukin (IL)-10 [8]. These effector and regulatory B-cell functions contribute to both normal immune regulation and also immunopathology [7, 9]. Though immature, peripheral B cells during development have a distinguished role in immune responses apart from the mature B cells. They elicit T cell-independent rapid antibody responses to polysaccharides, lipids, and other non-protein antigens which cannot bind to major histocompatibility complex (MHC) molecules [10]. In mice, immature B cells with specialized functions were identified. Marginal zone (MZ) B cells and B-1 B cells known to elicit T cell-independent responses to antigens of microbes in mucosal tissues and microbes that enter peritoneum have been reported [11, 12]. Distinct IL-10-producing regulatory B cells (Bregs) with immature phenotype also have been recently identified in mice and also in humans [13, 14]. However, functions of peripheral immature B cells during normal immune responses are less well characterized and remain to be delineated in humans.

In this respect, pre-naïve B cells are an interesting human peripheral immature B-cell population worthy of further investigation. Pre-naïve B cells were phenotypically distinct from transitional and naïve B cells, expressing intermediate levels of CD38, CD10, CD9, and the ABCB1 transporter, and were also shown to be capable of differentiating into naïve B cells [3]. Pre-naïve B cells manifested a unique set of functional characteristics [3]. These cells had typical characteristics of immature B cells with shorter life span and defective responses to BCR stimulation. However, pre-naïve B cells were comparable to adult B cells in their capacity to respond to signaling through CD40. Moreover, collaboration with activated CD4^+^ T cells resulted in their differentiation into plasma cells with the secretion of Abs. Uniquely, pre-naïve B cells expressed CD5, a marker of the B-1 subset of murine B cells, which can function as a negative regulator of BCR signaling and promote maintenance of tolerance to auto-Ag [15–17]. Because of these unique functional characteristics, pre-naïve B cells have the potential to perform specialized functions during normal immune responses, not fully explored previously. In this study, we set out to determine the functional role of pre-naïve B cells during normal immune responses by activating pre-naïve B cells through CD40. In addition, we carried out experiments by using pre-naïve B cells from patients with systemic lupus erythematosus (SLE) to determine the role of these cells during pathologic immunity.

## Methods

### Isolation of human B and T cells

Blood samples were obtained from 50 healthy adult donors and from nine patients with SLE. Healthy individuals included 21 men and 29 women with a mean age of 29 years (range of 25–35 years). Individuals who were taking immunosuppressive drugs or who had a disease potentially affecting the immune system were excluded. Patients with SLE included one man and eight women with a mean age of 40 years (range of 25–64 years). All patients with SLE had active disease at the time of the blood sample collection (an SLE Disease Activity Index score of 12–26) and received low-dose glucocorticoid and hydroxychloroquine. One patient was taking mycofenolate mofetil in addition to the above treatment. All patients and healthy volunteers provided written informed consent prior to sample collection. This work was approved by the institutional review board of Ewha Womans University Hospital and the institutional review board of Seoul National University Hospital. Peripheral blood mononuclear cells were isolated by centrifugation on Biocoll Separating solution (Biochrom, Cambridge, UK), and B cells were enriched by using Rosette Sep human B-cell enrichment cocktails (StemCell Technology, Vancouver, BC, Canada). Enriched B cells were stained with a combination of anti-CD20-PerCP, anti-CD27-FITC, and anti-CD38-PE-Cy7 (all from BD Biosciences, Franklin Lakes, NJ, USA). Cells were sorted into CD20^+^CD27^−^CD38^lo^ naïve, CD20^+^CD27^−^CD38^Int^ pre-naïve, and CD20^+^CD27^+^ memory B cells by using a BD FACSAriaIII™ and very narrow gates centered around the median CD38 fluorescence intensity of each B-cell subset. CD4^+^CD25^−^ T cells were sorted after staining with a combination of anti-CD4-APC-Cy7 and anti-CD25-PE.

### Cell culture

Human CD154 expressing L cells (CD154-L cells, kindly provided by Rizgar A. Mageed, Queen Mary University of London) were cultured in Dulbecco’s modified Eagle’s medium containing 5 % fetal bovine serum (FBS) (Life Technologies, Carlsbad, CA, USA). The expression of CD154 was routinely checked by flow cytometry. Purified human T and B cells were co-cultured in RPMI 1640 containing 10 % FBS (Life Technologies) in 96-well plates at 3 × 10^4^ cells/200 μl. For stimulating B cells, CD154-L cells were cultured for 18 hours and were irradiated with 60 Gy and then co-cultured with sorted B cells.

### Cell functional assays

To measure IL-10 production by B cells, CD154-L cells were co-cultured with sorted B cells for 3 days and with 50 ng/ml phorbol-12-myristate-13-acetate (PMA) (Sigma-Aldrich, St. Louis, MO, USA), 1 μg/ml ionomycin (Sigma-Aldrich), and Golgistop (BD Biosciences) in the last 6 hours of culture. Cells were fixed, permeabilized, and stained with anti-human CD19-PE, IL-10-Alexa647, interferon-gamma (IFN-γ)-FITC monoclonal Abs or isotype controls. Intracellular cytokine was assayed by using a flow cytometer. Alternatively, the amount of IL-10 was measured in the culture supernatants before PMA, ionomycin, and Golgistop by enzyme-linked immunosorbent assay (ELISA) (R&D Systems, Minneapolis, MN, USA). To measure the influence of activated B cells on CD4^+^ T-cell activation, CD154-L cells were co-cultured with sorted CD4^+^CD25^−^ T cells and B cells at a ratio of 1:10:10 (CD154-L cells: B cells: effector T cells) in the presence of suboptimal stimulation with soluble anti-CD3 (5 μg/ml) and anti-CD28 (10 μg/ml) for 5 days. Sorted autologous effector T cells were labeled with 3 μM carboxyfluorescein diacetate (Life Technologies) before co-culture with B cells. Proliferation of T cells by CFSE (carboxyfluorescein succinimidyl ester) dilution was assayed by flow cytometry. The same culture condition was used to measure T-cell cytokine production. Intracellular IFN-γ and tumor necrosis factor-alpha (TNF-α) and the cytokines in the culture supernatants were measured by same methods employed in IL-10 measurement. For identification of CD4^+^ T cells, T cells were stained with anti-CD3-APC, as CD4 is downregulated after activation. To measure the influence of activated B cells on CD4^+^FoxP3^+^ T-cell differentiation, CD154-L cells were co-cultured with sorted CD4^+^CD25^−^ T cells and B cells at a ratio of 1:10:10 (CD154-L cells: B cells: effector T cells) in the presence of optimal stimulation with anti-CD3/CD28-coated beads at a ratio of 1:320 (beads: T cells). For identification of CD4^+^FoxP3^+^ T cells, T cells were stained with anti-CD3-APC and FoxP3-FITC. To induce in vitro differentiation of B cells into plasma cells, sorted B cells were co-cultured with 5 Gy-irradiated autologous effector T cells at a ratio of 1:1 in a 96-well round-bottom tissue culture plate pre-coated with 10 μg/ml anti-CD3 Abs (clone OKT3, eBioscience, San Diego, CA, USA) in the presence of soluble anti-CD28 Abs (5 μg/ml, clone CD28.2, eBioscience) in 96-well round-bottom tissue culture plates for 11 days. In some experiments, B cells were stimulated with 200 ng/ml IL-21 (Life Technologies) and 2 μg/ml anti-CD40 (R&D Systems) to induce differentiation in the presence or absence of 10 μg/ml anti-IL-10 (clone JES3-9D7, BD Biosciences). For analysis of accessory molecule expression, B cells were cultured in the presence of CD154-L cells for up to 2 days, and CD80, CD86, CD54, human leukocyte antigen (HLA)-DR expression was determined by flow cytometry after staining the cells with monoclonal Ab anti-CD80-PE, anti-CD86-PE, anti-HLA-DR-V450, or anti-CD54-PE. Recombinant human IL-10 (100 ng/ml, R&D Systems), anti-IL-10 (10 μg/ml, BD Biosciences), and anti-IL-10Rα (100 ng/ml, R&D Systems) blocking Abs were added where indicated. The amounts of IL-6 and TNF-α in the culture supernatants were analyzed by multiplex assay with Luminex (Bio-Rad Laboratories, Hercules, CA, USA).

### Flow cytometry

Cells were acquired on an LSRII flow cytometer (BD Biosciences), and data were analyzed by using FlowJo 9.6 software (Tree Star Inc., now part of FlowJo, LLC, Ashland, OR, USA).

### Measurement of antibody reactivity

Cell culture supernatants were screened for Ab reactivity to single-stranded DNA (ssDNA), double-stranded DNA (dsDNA), and histone by ELISA. All samples were diluted (1/2) with dilution buffer (2 % bovine serum albumin, 3 mM EDTA, 0.05 % Tween 20, and 0.1 % gelatin, all from Sigma-Aldrich) and incubated overnight. Microwell plates were washed and incubated with a secondary alkaline phosphatase-labeled goat anti-human IgM Ab (Southern Biotechnology, Birmingham, AL, USA) overnight, followed by development with para-nitrophenyl phosphate (Sigma-Aldrich). Serially diluted sera from patients with SLE were used for preparing standard curves. All samples were analyzed in duplicate.

### Examination of immunoglobulin heavy chain complementary region 3

Single-cell polymerase chain reaction was used to amplify rearranged heavy chain Ig genes from genomic DNA as previously described [4]. Sequences were compared with germline Ig genes by using the web-based analysis program JoinSolver [18, 19] to examine IgH CDR3 (IgH CDR3) characteristics.

### Statistical analysis

All values are expressed as mean ± standard error of mean. Mean fluorescence intensity values were calculated as the geometric mean. Data were compared by using the paired two-tailed Student’s *t* test. *P* values of less than 0.05 were considered significant. All statistical analyses were performed by using GraphPad Prism 5.0 (GraphPad Software, Inc., La Jolla, CA, USA).

## Results

### IL-10^+^ B cells are enriched within the human CD27^−^CD38^int^ pre-naïve B-cell population following CD40 engagement

**Fig. 1:**
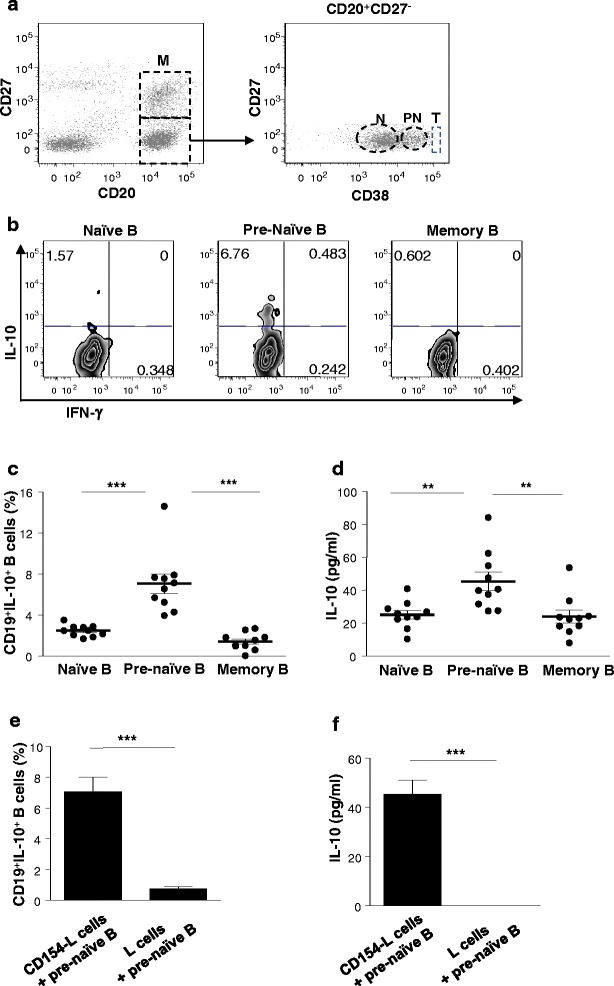
Identification of CD40-activated human B cells which produce high levels of interleukin-10 (IL-10). **a** Peripheral blood CD20^+^ B cells were stained for CD20, CD27, and CD38 and gated as previously shown [3]. A representative dot plot shows the CD27^−^CD38^lo^ naïve (N), CD27^−^CD38^Int^ pre-naïve (PN), CD27^−^CD38^hi^ transitional (T), and CD27^+^ memory (M) B-cell subsets (*n* = 50). **b** Representative contour plot showing the frequencies of IL-10-producing B cells measured by intracellular cytokine staining of naïve, pre-naïve, and memory B-cell populations following stimulation with CD154-L cells for 72 hours. **c** The frequencies of IL-10-producing B cells in B-cell subsets as described in (b) (*n* = 10, cumulative data from nine independent experiments). **d** Supernatants from cultures as described in (b) and (c) were tested for the presence of IL-10 by enzyme-linked immunosorbent assay (ELISA). **e** Frequencies of IL-10-producing pre-naïve B cells following stimulation with L cells with and without CD154 expression as described in (b) (*n* = 3, cumulative data from three independent experiments). **f** IL-10 production by pre-naïve B cells following stimulation with L cells with and without CD154 expression was measured from culture supernatants by ELISA as described in (e). **P < 0.01, ***P < 0.001. *IFN-γ* interferon-gamma, *IL* interleukin

To investigate whether pre-naïve B cells uniquely produce IL-10, B cells were stained to identify mature naïve (CD27^−^CD38^lo^), immature pre-naïve (CD27^−^CD38^int^), transitional (CD27^−^CD38^hi^), and memory (CD27^+^) subsets as previously shown (Fig. 1a) [3], and B-cell populations were assessed for IL-10 production after in vitro stimulation by engaging CD40. The frequency of IL-10^+^ B cells and IL-10 levels were significantly higher in the pre-naïve B-cell population compared with naïve B cells and memory B cells (Fig. 1b, c, d). Stimulation of pre-naïve B cells with CD154 was necessary to induce IL-10 production by the pre-naïve B cells, as unstimulated cells did not produce IL-10 (Fig. 1e, f).

### IL-10^+^ pre-naïve B cells did not suppress cytokine production by CD4^+^ T cells but were ineffective accessory cells for CD4^+^ T-cell activation and promote development of CD4^+^FoxP3^+^ T cells via IL-10

**Fig. 2:**
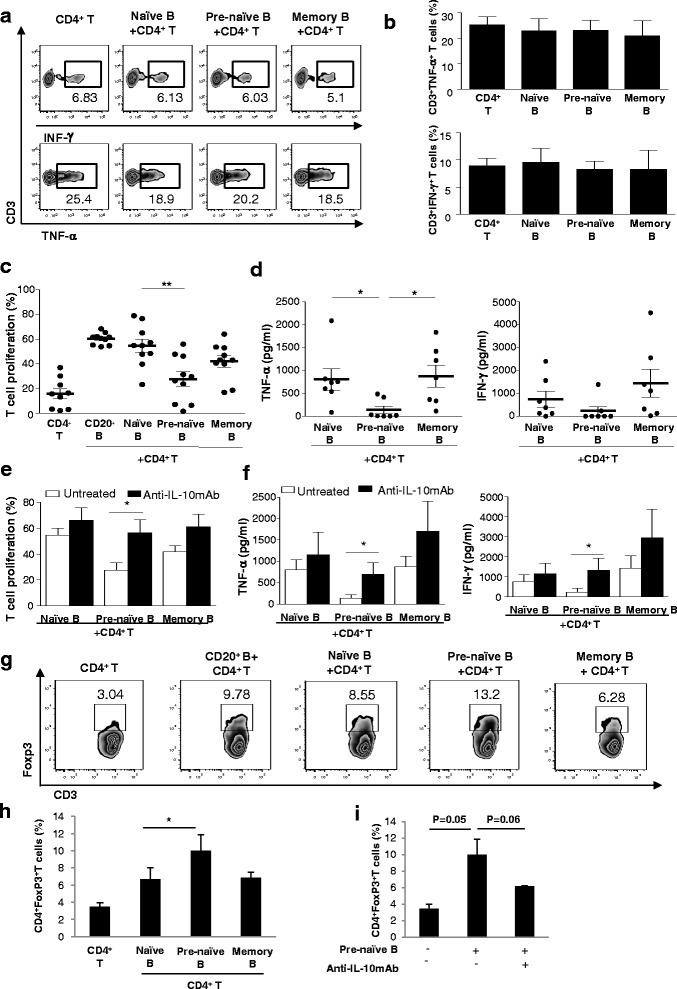
Activated pre-naïve B cells show IL-10-mediated ineffective promotion of CD4^+^ T-cell proliferation. **a** Representative contour plot showing the frequencies of CD4^+^INF-γ^+^ T cells and CD4^+^TNF-α^+^ T cells measured by intracellular cytokine staining after co-culture with B cells and CD154-L cells for 72 hours. **b** The frequencies of CD4^+^INF-γ^+^ T cells and CD4^+^TNF-α^+^ T cells measured as described in (a) (*n* = 4, cumulative data from three independent experiments). **c** Effect of total B cells and each B-cell subset on CD4^+^ T-cell proliferation in co-culture with B cells and CD154-L cells for 5 days (*n* = 10, cumulative data from eight independent experiments). **d** IFN-γ and TNF-α secretion was measured by enzyme-linked immunosorbent assay in the culture supernatants from experiments described in (c). **e** The effect of each B-cell subset on T-cell proliferation cells in co-culture of CD4^+^ T cells, B cells, and CD154-L cells for 5 days cultured in medium alone or in the presence of anti-IL-10 mAb (*n* = 6, cumulative data from five independent experiments). **f** IFN-γ and TNF-α secretion tested in supernatants from cultures as described in (e). **g** Representative histogram showing frequencies of CD4^+^FoxP3^+^ T cells in co-culture with CD4^+^ T cells, B cells, and CD154-L cells for 5 days. **h** The effect of each B-cell subset on frequencies of CD4^+^FoxP3^+^ T cells from experiments described in (g) (*n* = 3, cumulative data from three independent experiments). **i** The effect of pre-naïve B cells on frequencies of CD4^+^FoxP3^+^ T cells from experiments described in (g) cultured in medium alone or in the presence of anti-IL-10 mAb (*n* = 3, cumulative data from three independent experiments). *P < 0.05, **P < 0.01. *IFN-γ* interferon-gamma, *IL* interleukin, *mAb* monoclonal antibody, *TNF-α* tumor necrosis factor-alpha

We next determined whether IL-10^+^ pre-naïve B cells could regulate CD4^+^ T-cell cytokine production. CD4^+^ T cells were stimulated with anti-CD3 and anti-CD28, and the frequencies of CD4^+^INF-γ^+^ T cells and CD4^+^TNF-α^+^ T cells were measured by flow cytometry after 72 hours of co-culture with B cells stimulated with CD154-L cells. CD4^+^ T cells cultured alone or with naïve, pre-naïve, and memory B cells all differentiated into INF-γ^+^- and TNF-α^+^-producing cells at comparable frequencies. An inhibitory effect of IL-10^+^ pre-naïve B cells on CD4^+^ T-cell cytokine production was not observed compared with the naïve and memory B cells (Fig. 2a, b).

Next, the influence of IL-10^+^ pre-naïve B cells on CD4^+^ T-cell activation was determined. We used an in vitro T-cell and B-cell co-culture system employing a suboptimal T-cell activation signal (stimulation with soluble anti-CD3, 5 μg/ml and soluble anti-CD28, 10 μg/ml) so that effective T-cell proliferation was observed only after interaction with CD40-activated B cells. In this co-culture, T-cell proliferation was noted only when activated B cells were present. Naïve and memory B cells were both effective at promoting activated CD4^+^ T-cell proliferation, whereas pre-naïve B cells were less effective. In no circumstance did pre-naïve B cells depress CD4^+^ T-cell proliferation below that manifested in the absence of co-culture, but enhancement of CD4^+^ T-cell proliferation was deficient compared with naïve and memory B cells (Fig. 2c). In addition to their inability to promote optimal CD4^+^ T-cell proliferation, pre-naïve B cells were ineffective at supporting IFN-γ and TNF-α secretion by activated CD4^+^ T cells (Fig. 2d). To determine whether defective promotion of CD4^+^ T-cell activation was mediated by IL-10 produced by the activated pre-naïve B cells, CD4^+^ T-cell stimulation was carried out in the presence of a blocking anti-IL-10 mAb. Neutralizing IL-10 resulted in full recovery of the capacity of pre-naïve B cells to promote CD4^+^ T-cell proliferation to the level noted with the other B-cell subsets (Fig. 2e). In addition, blocking IL-10 resulted in enhancement of cytokine production by CD4^+^ T cells (Fig. 2f).

A previous report showed that human CD19^+^CD24^hi^CD38^hi^ Bregs induced the development of CD4^+^FoxP3^+^ Tregs and this was partially mediated by IL-10 [20]. To evaluate whether IL-10-mediated defective promotion of CD4^+^ T-cell activation by the activated pre-naïve B cells was associated with differentiation of Tregs, CD40-activated B cells were co-cultured with CD4^+^CD25^−^ T cells and stimulated with CD3/CD28-coated beads for 5 days and frequencies of CD4^+^FoxP3^+^ T cells were assessed. When CD4^+^ T cells were co-cultured with activated pre-naïve B cells but not with naïve or memory B cells, there was an increase in FoxP3-expressing CD4^+^ T cells (Fig. 2g, h). Neutralizing IL-10 in CD4^+^ T cell/activated pre-naïve B-cell co-culture resulted in reduced conversion of CD4^+^ T cells into CD4^+^FoxP3^+^ T cells to the level noted in co-cultures with the other B-cell subsets (Fig. 2i).

### Impaired expression of co-stimulatory molecules and secretion of lower amounts of cytokine involved in T-cell activation after CD40 signaling by pre-naïve B cells

**Fig. 3:**
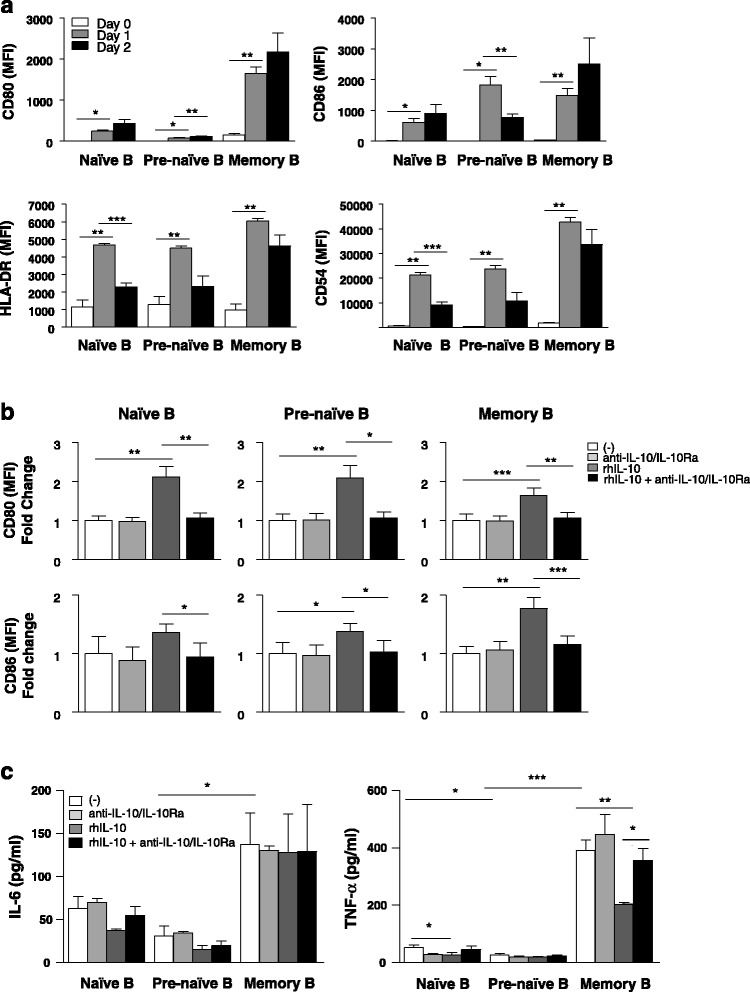
Impaired expression of co-stimulatory molecules and secretion of lower amounts of cytokine involved in T-cell activation by pre-naïve B cells. **a** Mean fluorescence intensity (MFI) of CD80, CD86, CD54, and HLA-DR expression of naïve, pre-naïve, and memory B-cell population on days 0–2 after stimulation with CD154-L cells (*n* = 18, cumulative data from 10 independent experiments). Data are expressed as mean MFI ± standard error (SE). **b** MFI of CD80 and CD86 expression of each B-cell population on day 1 of cultures described in (a) in medium alone or in the presence of anti-IL-10+ anti-IL-10Ra, rhIL-10, and rhIL-10+ anti-IL-10+ anti-IL-10Ra (*n* = 18, cumulative data from 10 independent experiments). Data are expressed as mean ± SE fold change over non-treated samples. **c** IL-6 and TNF-α levels in culture supernatants on day 1 of culture as described in (b). IL-6 and TNF-α secretion in the culture supernatants was measured by multiplex assay (*n* = 18, cumulative data from 10 independent experiments). Data are expressed as mean ± SE. *P < 0.05, **P < 0.01. ***P <0.001. *IL-10* interleukin-10, *TNF-α* tumor necrosis factor-alpha, *rhIL-10* recombinant IL-10, *anti-IL-10Ra* anti-IL-10 receptor antibody

To determine whether accessory molecule expression on pre-naïve B cells is impaired, B-cell populations were activated with CD154-L cells, and accessory molecule expression on each B-cell population upon activation was determined (Fig. 3a). Increase in expression of CD80, CD86, CD54, and HLA-DR was found on all three B-cell populations after CD40 stimulation for 1 day. Of note, pre-naïve B cells expressed a lower level of CD80 compared with naïve and memory B cells after CD40 stimulation, whereas CD86 expression was comparable to the level of expression on naïve and memory B cells. On day 2 of CD40 stimulation, all three B-cell populations increased CD80 expression, although the level expressed by pre-naïve B cells remained substantially lower than that of the other B-cell populations. CD86 expression was also upregulated between 24 and 48 hours by naïve and memory B cells, whereas pre-naïve B cells downregulated CD86 expression on day 2 of CD40 stimulation. CD54 and HLA-DR expression was comparable on all three B-cell populations. Blocking with IL-10/IL-10 receptor Abs did not increase co-stimulatory molecule expression by any B-cell population. However, IL-10 enhanced CD80 and CD86 expression on CD40-activated B cells and this effect was reversed with blocking IL-10/IL-10 receptor Abs (Fig. 3b).

To examine the effects of secretion of cytokines involved in T-cell activation, we measured IL-6 and TNF-α produced by the B cells after CD40 stimulation (Fig. 3c). We found that pre-naïve B cells produced significantly lower levels of TNF-α (27.5 ± 22.7 pg/ml) compared with memory (391.3 ± 73.3 pg/ml, *P* < 0.001) and naïve (52.1 ± 17.5 pg/ml, *P* = 0.027) B cells and produced significantly lower levels of IL-6 (30.1 ± 22.7 pg/ml) compared with memory B cells (137.53 ± 73 pg/ml, *P* = 0.015). Blocking with IL-10/IL-10 receptor Abs did not affect the secreted levels of IL-6 and TNF-α in any B-cell population. However, addition of IL-10 inhibited TNF-α production by naïve and memory B cells but had minimal effect on pre-naïve B cells. The effect of IL-10 on TNF-α production by memory B cells was reversed by blocking with IL-10/IL-10 receptor Abs.

### Antibodies produced by pre-naïve B cells are autoreactive

**Fig. 4:**
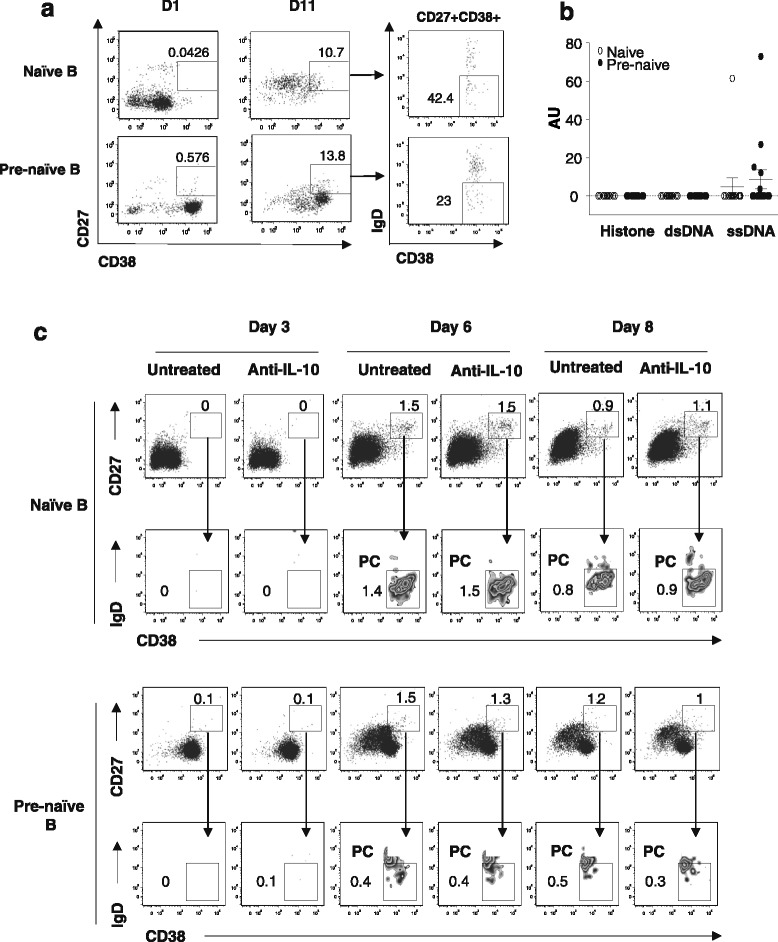
Antibodies produced by pre-naïve B cells are autoreactive. **a** Sorted pre-naïve and naïve B cells were cultured with irradiated CD4^+^ T cells in anti-CD3 pre-coated microtiter wells and, after 11 days of culture, were assessed for CD38^high^CD27^+^IgD^−^ plasma cells. Numbers represent percentage of CD27^+^CD38^high^ B cells and CD27^+^CD38^high^IgD^−^ B cells. **b** Reactivity of antibodies produced by pre-naïve and naïve B cells. Culture supernatants from experiment described in (a) from pre-naïve B cells (●) and naïve B cells (○) were analyzed for reactivity with indicated antigens, histone, dsDNA, and ssDNA by enzyme-linked immunosorbent assay. Values indicate results from individual donors of a total of 15 tested. **c** Sorted pre-naïve and naïve B cells were stimulated with anti-CD40 2 μg/ml and interleukin-21 (IL-21) 200 ng/ml in the presence and absence of anti-IL-10 monoclonal antibodies 10 μg/ml and assessed for frequencies of CD27^+^CD38^high^IgD^−^ plasma cells at days 3, 6, and 8. Numbers represent percentage of cells. *A.U.* arbitary units, *dsDNA* double-stranded DNA, *PC* plasma cells, *ssDNA*, single-stranded DNA

Stimulated immature B cells produce predominantly autoreactive Abs [21]. We cultured pre-naïve B cells with optimally activated CD4^+^ T cells to induce differentiation into Ab-secreting plasma cells to test whether secreted Abs are autoreactive. As can be seen, a large population of CD27^+^CD38^high^ plasma cells was generated (Fig. 4a). As a measure of self-reactivity, we tested IgM Abs for binding to a defined set of Ags, including ssDNA, dsDNA, and histone, by ELISA in culture supernatants from 10 healthy donors. We found that IgM Abs produced by pre-naïve B cells from 5 out of 10 donors were reactive to ssDNA but that only 1 out of 10 naïve B-cell cultures was reactive to anti-ssDNA (Fig. 4b). Anti-histone or anti-DNA was produced by neither naïve nor pre-naïve B cells in any of the donors tested. Importantly, neutralization of IL-10 had no effect on the capacity of pre-naïve B cells to differentiate into plasma cells (Fig. 4c).

Since previous analysis of human Ab sequences showed that long IgH CDR3 or highly positively charged IgH CDR3s (or both) were associated with self-reactivity of the Abs [22, 23], we examined characteristics of the IgH CDR3 from transitional, pre-naïve, and naïve B-cell populations (Table 1 and Additional file 1: Figure S1). By comparing the distribution between the productive and the non-productive repertoires of transitional, pre-naïve, and naïve B cells, we found that negative selection for long and highly positive charged IgH CDR3 occurred in all three B-cell populations. However, analysis of the productive repertoires revealed that CDR3_H_ of pre-naïve B cells (52.5 ± 12.5 bp) was significantly longer compared with that of the transitional (48.9 ± 12.6 bp) and the naïve (49.0 ± 10.5 bp) B cells (*P* <0.01). This skewing for longer CDR3_H_ length in pre-naïve B cells was related to maintenance of significantly longer germline D segment.

**Table 1:** Characteristics of immunoglobulin heavy chain CDR3 from peripheral blood transitional, pre-naïve, and naïve B-cell populations

	Number of sequences	Mean CDR3_H_ length (nucleotides ± SD)	Mean D length (nucleotides ± SD)
Productive rearrangements			
Transitional B	109	48.9 ± 12.6	14.7 ± 5.1
Pre-naïve B	200	52.5 ± 12.5^a,b^	17.3 ± 5.8
Naïve B	146	49.0 ± 10.5	15.9 ± 5.2
Non-productive rearrangements			
Transitional B	24	57.3 ± 10.9^c^	18.4 ± 5.7
Pre-naïve B	71	59.9 ± 16.5^c^	18.4 ± 6.0
Naïve B	39	60.3 ± 16.3^c^	18.4 ± 5.9

*SD* standard deviation

^a^Significant (*P* < 0.05) difference between transitional and pre-naïve B cells

^b^Significant (*P* < 0.05) difference between pre-naïve and naïve B cells

^c^Significant (*P* < 0.01) between the productive and non-productive rearrangements

### SLE pre-naïve B cells differ from normal B cells in IL-10 production and co-stimulatory molecule expression

**Fig. 5:**
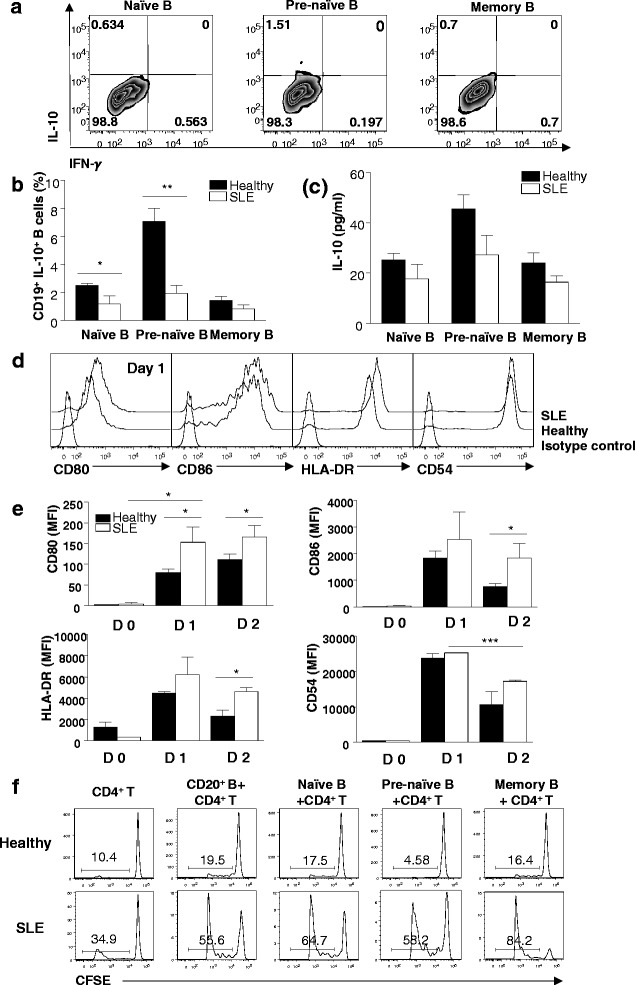
Systemic lupus erythematosus (SLE) pre-naïve B cells were defective in producing interleukin-10 (IL-10) and promoted robust CD4^+^ T-cell activation. **a** Representative contour plot showing the frequencies of IL-10-producing B cells from an SLE patient, measured as described in Fig. 1b. **b** Comparison of the frequencies of IL-10-producing B cells between six patients with SLE and 10 healthy donors (cumulative data from 6 independent experiments). **c** Supernatants from cultures as described in (b) were tested for the presence of IL-10 by enzyme-linked immunosorbent assay. **d** Representative histogram comparing CD80, CD86, CD54, and HLA-DR expression on pre-naïve B cells from a healthy individual and an SLE patient, obtained as described in Fig. 3a. **e** Comparison of mean fluorescence intensity of CD80, CD86, CD54, and HLA-DR expression of pre-naïve B cells from six healthy individuals and three SLE patients on days 0–2 (cumulative data from three independent experiments). Data are expressed as mean ± standard error. **f** Representative histogram showing proliferation of T cells in co-culture with B cells from two SLE patients and two healthy donors. Proliferation of T cells was determined by carboxyfluorescein succinimidyl ester (CFSE) dilution. *P < 0.05, **P < 0.01, ***P < 0.001. IFN-γ interferon-gamma, *MFI* mean fluorescence intensity

Because SLE is associated with loss of tolerance, we examined whether regulatory activities of pre-naïve B cells are altered in patients with SLE. In contrast to pre-naïve B cells from healthy donors, SLE pre-naïve B cells failed to produce IL-10 upon stimulation with CD154-L cells (Fig. 5a). In SLE, frequencies of IL-10^+^ B cells and levels of IL-10 production were low, similar to those of naïve and memory B-cell populations (Fig. 5b, c).

Next, we evaluated whether the accessory molecule expression pattern was different in pre-naïve B cells from the SLE patients compared with the healthy individuals after CD40 stimulation (Fig. 5d, e). An increase in expression of CD80 was found by SLE pre-naïve B cells, compared with healthy pre-naïve B cells, after CD40 stimulation for 1 day, whereas CD86, HLA-DR, CD54 expression was not significantly different. On day 2 of CD40 stimulation, early downregulation of CD86 and HLA-DR expression found in healthy pre-naïve B cells was not found in SLE pre-naïve B cells. SLE pre-naïve B cells continued to maintain their expression levels of CD86 and HLA-DR for up to day 2 of CD40 stimulation. In addition, we found that SLE B cells were more effective in stimulating CD4^+^ T-cell activation compared with the healthy individuals. Of note, SLE pre-naïve B cells promoted CD4^+^ T-cell proliferation comparably to naïve and memory B-cell (Fig. 5f).

## Discussion

Pre-naïve B cells are a unique intermediate maturational subset with some immunocompetent functions, but their role in immune regulation has not been previously defined. In this study, we demonstrated two distinct functions of human peripheral pre-naïve B cells during normal immune responses: production of autoreactive Abs and maintenance of peripheral tolerance by auto-regulation of their accessory cell function.

B cells with autoreactive BCRs are constantly generated by random Ig gene rearrangement during B-cell ontogeny. It was reported in humans that approximately half of all BCRs expressed by immature B cells are autoreactive [21]. Since pre-naïve B cells are developmentally not fully mature, it was possible that Abs produced by the pre-naïve B cells were autoreactive. In accordance with previous findings, we found that IgM Abs produced by pre-naïve B cells from 50 % of the normal donors tested were reactive to ssDNA and had longer IgH CDR3 length compared with transitional and naïve B cells suggestive of self-reactivity [22, 23]. Though paradoxical to B-cell tolerance, auto-Abs are known to perform some useful functions during immune responses. An example of such beneficial auto-Abs are natural Abs, which are shown to be an essential first-line defense against pathogens [24] and are shown to participate in the clearances of apoptotic cells [25]. In mice, natural Abs are secreted mainly by a distinct B-cell subset, B-1 cells [15], but in humans, existence of a distinct B-cell subset similar to mouse B-1 cells is less clear, and natural Abs are produced mainly by CD5^+^ B cells not committed to a distinct lineage. Fetal and neonatal B cells [26, 27] as well as CD5^+^ B cells from healthy adults and from patients with chronic lymphocytic leukemia and autoimmune diseases such as rheumatoid arthritis and SLE were shown to produce natural IgM autoreactive Abs [28–30]. Autoreactivity of Abs produced by the pre-naïve B cells and the fact that these cells are CD5^+^ suggest that pre-naïve B cells may be one source of natural Abs in adult humans functioning to clear apoptotic material and other auto-Ags or to provide primary defense against certain bacterial and viral pathogens during humoral immune response.

To maintain tolerance, most of the autoreactive immature B cells in the periphery are culled during development before they enter the mature B-cell compartment by a mechanism of deletion, since they are highly susceptible to deletion by receptor cross-linking [31]. Because pre-naïve B cells may contribute to protective autoreactive Ab production during normal immune response, it would be a loss if these cells were completely deleted from the repertoire. Their presence poses a risk, however, if they could function as Ag-presenting cells (APCs). The presence of a BCR with specificity for auto-Ags might permit them to present auto-Ags to CD4^+^ T cells. We found unique mechanisms to limit this possibility in healthy individuals. The first mechanism employed by the pre-naïve B cells is regulation of co-stimulatory molecules upon receiving CD40 signal. We found that CD40-activated pre-naïve B cells inherently displayed lower levels of CD80 and that the more widely and predominantly displayed CD86 was displayed only transiently but that naïve and memory B cells continued to upregulate both CD80 and CD86. CD80 and CD86 are well-defined potent co-stimulators that play a major role in the activation of T cells by binding to CD28 [32]. It has been suggested that CD86 is more important than CD80 in mediating resting T-cell activation by APCs [33] and is essential for autoreactive T-cell activation and development for autoimmunity [34]. Early downregulation of CD86 by pre-naïve B cells may be one of the mechanisms to block the autoimmune response from occurring by chance. Of note, this mechanism appeared to be independent of IL-10 secretion, even though IL-10 has been shown to regulate co-stimulatory molecule expression in dendritic cells (DCs) and macrophages by means of the E3 ubiquitin ligase membrane-associated ring-CH 1 (MARCH 1) [35, 36].

A second mechanism to limit stimulation of autoreactive CD4^+^ T cells is through regulation of cytokine secretion. IL-10 is one of the most important anti-inflammatory cytokines regulating T-cell responses produced by macrophages, DCs, B cells, and various subsets of CD4^+^ and CD8^+^ T cells [37]. IL-10 produced by the B cells is known to mediate immunosuppression and to regulate T-cell responses directly or by induction of Tregs [20, 38–40]. However, the capacity of IL-10 produced by pre-naïve B cells to downregulate the ability of these cells to augment CD4^+^ T-cell activation has not been previously reported. We found that pre-naïve B cells produced IL-10 upon receiving a CD40 signal and that IL-10 prevented these cells from maximally supporting the stimulation of CD4^+^ T-cell proliferation and biased these cells away from supporting T-cell proliferation toward participation in the differentiation of CD4^+^ T cells into CD4^+^FoxP3^+^ Tregs. We are unable to fully explain the endocrine effect of secreted IL-10 on pre-naïve B-cell co-stimulatory function. In DCs and monocytes, downregulation of CD86 [41, 42] and MHC class II molecules [43, 44] was reported to be involved in IL-10-mediated poor support of T-cell responses. In contrast, it was also reported that IL-10 activates B cells and upregulates MHC class II molecule [45, 46]. We also found that IL-10 mediated modest upregulation of CD80 and CD86, suggesting that other mechanisms employed by IL-10 to mediate poor support of T-cell activation exist which counteract B-cell activation by IL-10. In addition to production of immunoregulatory cytokine IL-10, pre-naïve B cells secreted significantly lower amounts of T-cell activating cytokines IL-6 and TNF-α, which can further prevent these cells from promoting T-cell activation.

Thus far, IL-10-producing B cells exhibiting regulatory functions independent of secreted IgG have been classified as Bregs in both mouse and humans [8]. Stimulation with a combination of Ag, CD154, and Toll-like receptor ligands was reported to induce production of IL-10 by the Bregs [8]. Phenotypically, two subsets of human IL-10-producing Bregs were described: CD19^+^CD24^high^CD38^high^ immature transitional B cells and CD19^+^CD24^hi^CD27^+^ memory B cells [14, 47]. Pre-naïve B cells also produce IL-10 after CD40 stimulation but are not classic Bregs phenotypically. They are immature B cells and express intermediate levels of CD38 and CD24 compared with the CD19^+^CD24^high^CD38^high^ Breg cells described previously [3]. In addition, pre-naïve B cells appear to regulate immune responses through limiting their own Ag-presenting function, not by directly suppressing CD4^+^ T cells as with Bregs. Therefore, IL-10 produced by the pre-naïve B cells may contribute to maintenance of self-tolerance by limiting the capacity to activate CD4^+^ T cells rather than by directly suppressing their function.

Patients with SLE have been suggested to have a defect in early B-cell tolerance checkpoints leading to the accumulation of a large number of naïve B cells that express BCRs that recognize self-Ags, even in the inactive phase of the disease [48]. Consistent with this finding, Yurasov et al. have demonstrated that 25–50 % of the naïve B cells in patients with SLE produced self-reactive Abs even before they participate in immune responses [49]. However, the precise mechanisms contributing to early tolerance defects have not been reported until recently. In a recent study, CD19^+^CD24^hi^CD38^hi^ Bregs from patients with SLE were found to be refractory to CD40 engagement and produced less IL-10 and lacked CD4^+^ T-cell suppressive capacity compared with the healthy controls, suggesting that functional defects of Bregs could lead to autoimmunity [14]. We found that pre-naïve B cells from patients with SLE also showed a defect in producing IL-10 following CD40 stimulation and that their limited co-stimulatory molecule expression upon CD40 signaling was also impaired. SLE pre-naïve B cells expressed higher levels of CD80, CD86, and HLA-DR after CD40 stimulation and effectively promoted CD4^+^ T-cell activation compared with the healthy controls. Since IL-10 and deficient co-stimulatory molecule expression appear to be a novel mechanism of self-tolerance employed by the pre-naïve B cells, a defect in pre-naïve B-cell functions could lead to a break in self-tolerance, especially in the SLE milieu, where expansion of the pre-naïve B cells has been found [3, 4], and the immune system is intensely activated as evidenced by elevated B cell-activating factor (BAFF) levels, hyperexpression of CD40L on T cells, and/or increased levels of certain cytokines such as IL-21 [50–53]. In mice, BAFF has been shown to increase IL-10 production from MZ B cells [54]. The relevance of this to human B cells is unknown since pre-naïve B cells respond poorly to BAFF [3], and many patients with SLE have increased levels of BAFF but decreased IL-10 production [55, 56]. It is intriguing to note that SLE pre-naïve B cells may play a unique role in activating autoimmune CD4^+^T cells since they have enhanced co-stimulatory capacity and also more frequently express autoreactive BCR. This combination of abnormalities may make them uniquely suited to take up and process auto-Ags and present the relevant peptide to autoreactive CD4^+^ T cells, thus contributing to the break in T-cell tolerance and the upregulation of autoimmunity in patients with SLE.

## Conclusions

Our data suggest that there are inherent and IL-10-dependent mechanisms that limit the capacity of activated pre-naïve B cells from participating in cellular immune response, but these cells can participate in humoral immune responses by production of auto-Abs. Defects in these pre-naïve B-cell mechanisms in SLE may contribute to a break in self-tolerance and could enhance the development of autoimmunity. Further understanding of the mechanisms underlying the functional defects of pre-naïve B cells in patients with SLE could provide insights into the pathogenesis of this complex systemic autoimmune disease.

## Additional file

Additional file 1: Figure S1. Characteristics of the immunoglobulin complementary region 3 (IgH CDR3) from transitional, pre-naïve, and naïve B-cell populations. (a) Proportion of positively charged amino acids and frequencies of IgH CDR3 length in productive repertoire of each B-cell population. **Significant difference (*P* < 0.01) between the productive and non-productive repertoires in IgH CDR3s with at least three positive charges. (b) Frequencies of IgH CDR3 length in productive repertoire of each B-cell population.

## Abbreviations

Ab: Antibody
Ag: Antigen
APC: Antigen-presenting cell
BAFF: B cell-activating factor
BCR: B-cell receptor
bp: Base pairs
Breg: Regulatory B cell
DC: Dendritic cell
dsDNA: Double-stranded DNA
ELISA: Enzyme-linked immunosorbent assay
FITC: Fluorescein isothiocyanate
HLA: Human leukocyte antigen
IFN: Interferon
Ig: Immunoglobulin
IgH CDR3: Immunoglobulin complementary region 3
IL: Interleukin
MHC: Major histocompatibility complex
MZ: Marginal zone
PMA: Phorbol-12-myristate-13-acetate
SLE: Systemic lupus erythematosus
ssDNA: Single-stranded DNA
TNF: Tumor necrosis factor
Treg: Regulatory T cell
