# Supplementary figures and images for: Autoregulatory function of interleukin-10-producing pre-naïve B cells is defective in systemic lupus erythematosus
Source: Arthritis Res Ther. 2015 Jul 25;17(1):190. doi: 10.1186/s13075-015-0687-1 (PMC4515025; doi:10.1186/s13075-015-0687-1)

Figure S1a

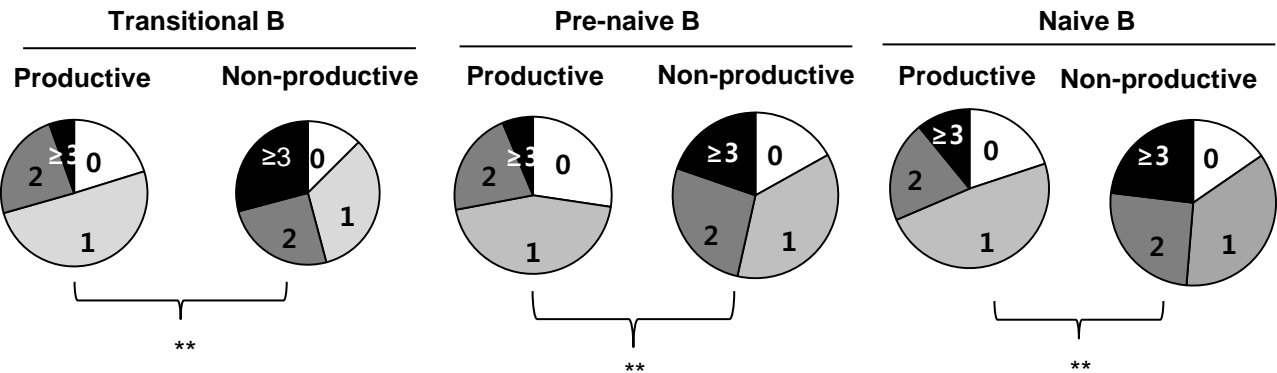

Figure S1b

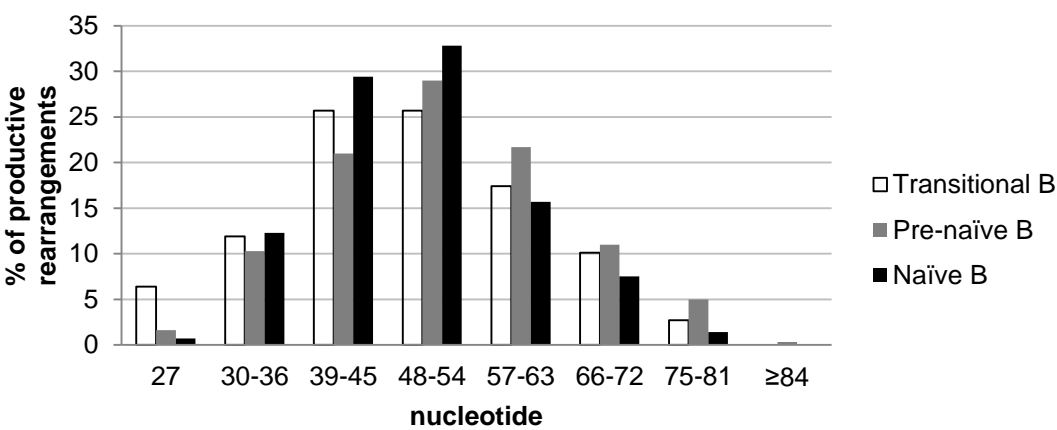

Supplement: Additional file 1: Figure S1. — Characteristics of the immunoglobulin complementary region 3 (IgH CDR3) from transitional, pre-naïve, and naïve B-cell populations. (a) Proportion of positively charged amino acids and frequencies of IgH CDR3 length in productive repertoire of each B-cell population. **Significant difference (P < 0.01) between the productive and non-productive repertoires in IgH CDR3s with at least three positive charges. (b) Frequencies of IgH CDR3 length in productive repertoire of each B-cell population. [file 13075_2015_687_MOESM1_ESM.pdf]
